# Economic Hardship and Life Expectancy in Nassau County, Florida

**DOI:** 10.5888/pcd16.180481

**Published:** 2019-03-14

**Authors:** Jessica Joiner, Melissa Jordan, Keshia Reid, Kristina Kintziger, Chris Duclos

**Affiliations:** 1 Florida Department of Health, Public Health Research Unit, Division of Community Health Promotion, Tallahassee, Florida; 2 University of Tennessee, Department of Public Health, Knoxville, Tennessee

**Figure:**
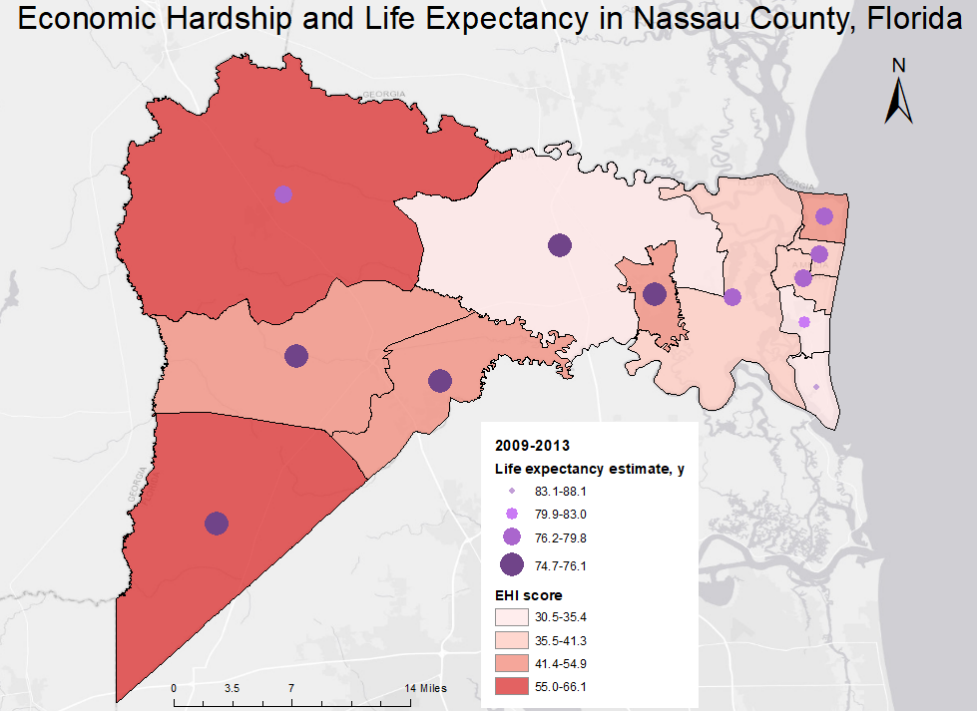
Economic Hardship Index (EHI) scores and life expectancy estimates at birth, by census tract, in Nassau County, Florida. EHI scores and life expectancy estimates were grouped into 4 classes by using Jenks natural breaks and combined to produce a bivariate map. EHI scores range from 0 to 100, with higher scores indicating worse economic conditions. The map highlights marked differences in economic conditions and life expectancy between census tracts. The map was shared with county health department staff members in response to calls for locally relevant data to address health disparities.

**Table:** 

Location in County	Life Expectancy Estimate, y	EHI Score
Northwest	77.5	55.0–66.1
Middle west	75.4	41.4–54.9
Middle west	75.2	41.4–54.9
Southwest	76.1	55.0–66.1
Central	75.5	30.5–35.4
Central	74.7	41.4–54.9
Central	77.1	35.5–41.3
North coast	79.3	41.4–54.9
Middle coast	78.6	35.5–41.3
Middle coast	79.8	35.5–41.3
South coast	83.0	30.5–35.4
South coast	88.1	30.5–35.4

## Background

The Economic Hardship Index (EHI) combines 6 social and economic measures to provide a more complete picture of socioeconomic conditions in a community than any one measure alone (1,2). It has the advantage of comprising data that operate at the community level, rather than the individual or family level, and it allows for a comparison of one community relative to another (or itself) over time (3). Socioeconomic conditions in a community are strongly associated with health (4). Economic hardship can affect a person’s ability to access important health care services and lead a healthy lifestyle. Local estimates of life expectancy are useful in understanding the contributions of socioeconomic conditions to population health (2). Life expectancy data allow the examination of health disparities by place, because it reflects the combined effect of major illnesses and injuries and their underlying causes, including social and environmental determinants of health (5).

In response to calls for relevant and timely community-level indicators that address underlying causes of illness and injury and advance health equity, the Florida Department of Health used the EHI to test a bivariate mapping technique that combined data on economic hardship and life expectancy at the census-tract level in Nassau County, Florida. Nassau County is in northeastern Florida along the Atlantic coast. It has a land area of 725.9 square miles and a population of 73,314 (6). The average population per census tract in Nassau County is 5,640 (6).

## Methods

We calculated the EHI by using 6 indicators from the US Census Bureau’s 2014 American Community Survey (ACS) 5-year estimates: unemployment (percentage of the population aged ≥16 who were unemployed), population dependency (percentage of the population aged <18 or >64), educational attainment (percentage of the population aged ≥25 with less than a high school diploma), per capita income, crowded housing (percentage of occupied housing units with >1 person per room), and poverty (percentage of persons living below the federal poverty level). We standardized these indicators within each tract to give them equal weight and combined them into a composite score. Scores range from 0 to 100, with higher scores indicating worse economic conditions. Additional methodologic details about the index are reported elsewhere (3).

We calculated life expectancy estimates by using 5 years (2009–2013) of aggregated mortality data geocoded to 2010 census tract areas. We used the adjusted Chiang II method to generate life expectancy estimates for all tracts (7). This method uses the life table approach and assumes that deaths are spread evenly throughout each age period. It also handles zero deaths in a given age category and is adjusted to account for variance in the last age interval — all of which are important considerations in calculating life expectancy estimates (7). We suppressed life expectancy estimates with a standard error of 2 years or more because of low numbers of deaths or small populations.

We used ArcMap 10.3.1 for Desktop (Esri) to join EHI scores and life expectancy estimates to the 2010 census tract shapefile and produce a bivariate map that displays life expectancy as graduated circles and EHI scores as a choropleth map. Each variable was categorized into 4 classes by using the Jenks natural breaks method. We performed correlation analysis in SAS version 9.4 (SAS Institute Inc).

## Findings

Average life expectancy in Nassau County was 77.9 years (95% confidence interval [CI], 77.4–78.5 y), comparable to the national average of 78.7 years (8). We observed a gap of approximately 13 years between the tract with the shortest life expectancy (74.7 [95% CI, 73.1–76.2] y) and the tract with the longest life expectancy (88.1 [95% CI, 84.8–91.5] y). EHI scores ranged from a low of 30.5 (least hardship) to a high of 66.1 (greatest hardship). Most tracts followed the expected pattern, such that areas with higher levels of economic hardship generally had lower life expectancy. Overall, tracts with the highest levels of economic hardship and lower life expectancy were concentrated on the eastern side of the county. The 2 tracts with the lowest level of economic hardship and highest life expectancy were along the coast and shared a boundary. A simple correlation analysis showed a moderate negative association between life expectancy and economic hardship (*r* = −0.494, *P* = .10), although this association was not significant because of the small sample size (n = 12).

## Action

The Florida Department of Health’s Health Equity Program Council (HEPC) Data and Assessment Subcommittee has piloted maps for several counties. These maps were shared with county health administrators and the state health department’s central office staff members who seek to link community-level data to Florida’s State Health Improvement Plan. Linking EHI scores with life expectancy estimates provides county health departments a more complete picture of neighborhood conditions than any one measure alone. The Nassau County map was distributed as part of a press release by the local health department and received news coverage in which residents shared their thoughts on the map and life expectancy disparities in their community. In the news story, community members expressed surprise at the disparities, but they also felt that areas with higher economic hardship were indeed areas of poorer health. We hope this publicity will expand beyond Nassau County and generate more interest in addressing health disparities by using locally relevant data and bivariate mapping techniques.

## References

[1] Chicago Department of Public Health, Epidemiology and Public Health Informatics. Selected socioeconomic indicators in Chicago, 2008–2012. https://data.cityofchicago.org/Health-Human-Services/Census-Data-Selected-socioeconomic-indicators-in-C/kn9c-c2s2. Accessed November 14, 2018.

[2] Boothe VL , Fierro LA , Laurent A , Shih M . Sub-county life expectancy: a tool to improve community health and advance health equity. Prev Chronic Dis 2018;15:E11. 10.5888/pcd15.170187 29369759 PMC5798219

[3] Montiel LM , Nathan RP , Wright DJ . An update on urban hardship. Albany (NY): The Nelson A. Rockefeller Institute of Government; 2004.

[4] Adler NE , Stewart J , Cohen S , Cullen M , Diez Roux AV , Dow W , Reaching for a healthier life: facts on socioeconomic status and health in the U.S. San Francisco (CA): The John D. and Catherine T. MacArthur Foundation Research Network on Socioeconomic Status and Health; 2007.

[5] Los Angeles County Department of Public Health, Office of Health Assessment and Epidemiology. Life expectancy in Los Angeles County: how long do we live and why? A cities and communities report. Los Angeles (CA); 2010.

[6] US Census Bureau. 2010 Census summary file 1: table GCT-PH1. https://factfinder.census.gov/faces/nav/jsf/pages/community_facts.xhtml. Accessed November 14, 2018.

[7] Chiang CL . A stochastic study of the life table and its applications. II. Sample variance of the observed expectation of life and other biometric functions. Hum Biol 1960;32:221–38. 13693002

[8] National Center for Health Statistics. Health, United States, 2016: with chartbook on long-term trends in health. Hyattsville (MD); National Center for Health Statistics; 2017. 28910066

